# Induction of cellulase production in *Trichoderma reesei* by a glucose–sophorose mixture as an inducer prepared using stevioside†

**DOI:** 10.1039/d2ra01192a

**Published:** 2022-06-13

**Authors:** Peng Zhang, Qian Li, Yudian Chen, Nian Peng, Wenshu Liu, Xuemei Wang, Yonghao Li

**Affiliations:** Chongqing Key Laboratory of Industrial Fermentation Microorganism, School of Chemistry and Chemical Engineering, Chongqing University of Science and Technology Chongqing 401331 China yh_li@cqust.edu.cn +86-23-65022211

## Abstract

Sophorose is currently the most effective inducer of cellulase production by *Trichoderma reesei*; however, the use of byproduct sophorose from the stevioside acid hydrolysis process has not been developed. In this study, stevioside was hydrolysed with different concentrations of HCl to obtain isosteviol and a mixture of glucose and sophorose (MGS). Isosteviol showed good inhibitory effects on the growth of *Aspergillus niger*, *Saccharomyces cerevisiae* and *Escherichia coli* after separation. At the same time, MGS, as a byproduct, was evaluated for cellulase production to determine the feasibility of this approach. MGS was compared with common soluble inducers, such as lactose, cellobiose, and a mixture of glucose and β-disaccharide (MGD), and induced higher cellulase production than the other inducers. The cellulase activity induced by MGS was 1.64- and 5.26-fold higher than that induced by lactose and cellobiose, respectively, and was not significantly different from that induced by MGD. The crude enzyme using MGS as an inducer with commercial β-glucosidase was further tested by hydrolyzing NaOH-pretreated corn stover with 5% solid loading, and 33.4 g L^−1^ glucose was released with a glucose yield of 96.04%. The strategy developed in this work will be beneficial for reducing inducer production cost through a simple stevia glycoside hydrolysis reaction and will contribute to studies aimed at improving cellulase production using soluble inducers for easier operation in industrial-scale cellulase production.

## Abstract 2

Inducing the synthesis of active cellulase is critical for biorefineries. The effective inducer was prepared from stevioside, which contains sophorose.

## Graphical abstract

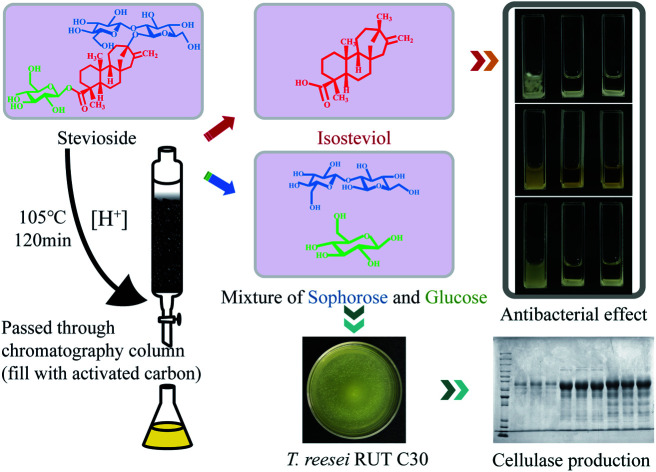

## Introduction

Lignocellulosic biorefineries, which involve the production of biofuels and biochemicals from forestry and agricultural residues, are important alternatives for addressing the energy crisis and for sustainable economic development.^1,2^ In recent studies, *Trichoderma reesei* was the main producer used for commercial lignocellulolytic enzyme preparation; however, the high cost of inducers and low induction efficiency are the major bottlenecks limiting the biorefining of lignocellulose.^3,4^

Using inducers is essential for cellulase production. The inducers of *T. reesei* can be roughly divided into two categories: solid inducers and soluble inducers.^5^ For solid inducers, which allow the fermentation liquor to be a serious non-Newtonian fluid and are not easy to feed during high-density fermentation, the heat transfer efficiency may be lower than that associated with soluble inducers. Moreover, during the fermentation induction process, a low level of basal cellulase from *T. reesei* is always required to hydrolyse solid inducers to produce small molecules, which can then enter cells to induce cellulase production; therefore, the induction efficiency of solid inducers is unsatisfactory, and the fermentation of *T. reesei* with solid inducers has no application prospects.^6–8^

For soluble inducers, the heat transfer efficiency in the fermenter is high, and energy consumption is low. Moreover, a higher induction efficiency can be achieved with soluble inducers because cellulase production can be directly induced in cells without the need for a basal cellulase level to degrade the inducer into small molecules.^9–11^ Lactose and sophorose are the most widely used soluble inducers. However, lactose is not suitable for the industrial production of cellulase due to its low productivity and high cost in China.^5,12,13^ On the other hand, the induction effect of sophorose is 2500 times higher than that of cellobiose,^14,15^ which is the most efficient cellulase inducer of *T. reesei* identified thus far. However, natural sophorose is relatively rare, so its cost remains high. In our previous studies, sophorose was produced from glucose through a transglycosylation reaction catalysed by β-glucosidase,^16^ which is an effective inducer of cellulase production in *T. reesei*. However, commercial β-glucosidase has to be used for the transglycosylation reaction, which increases the fermentation cost. In addition, sophorose production from glucose catalysed by β-glucosidase requires a long transglycosylation reaction (72 h), which makes the fermentation process extremely energy intensive, based on our current knowledge of this field.^17–19^ Sophorose and isosteviol were identified *via* the acid hydrolysis of stevioside as first described by Bridel and Lavieille in 1931.^20^

Stevioside is considered to be a natural noncaloric sweetener and is widely used in the business and catering industries.^21^ In addition to sweetness, stevia glycosides have various benefits, such as antihyperglycaemia, antihypertension, anti-inflammatory, antitumor and immunoregulatory effects.^22–24^ However, most studies tend to focus on isosteviol (*ent*-16-oxobeyran-19-oic acid), another product of hydrolysis, which is a sweet diterpenoid with a tetracyclic beyonderene-type skeleton. Isosteviol shows extensive bioactivity and is a high-value-added product.^25^ The different conjugated forms of isosteviol show anticancer activities and can thus be used for the synthesis of inexpensive chemotherapeutic agents.^26,27^ Moreover, research on isosteviol has revealed that it shows peculiar DNA polymerase, DNA topoisomerase inhibition and acetylcholine inhibition abilities and antidiarrheal and antibacterial activities.^28–30^ Another hydrolysate, sophorose, has not been directly applied for cellulase production by *T. reesei* to the best of our knowledge.

In this study, we focused on determining whether sophorose obtained *via* stevioside hydrolysis can be used in cellulase production. Sophorose and isosteviol were prepared by the acid hydrolysis of stevioside and separated in a chromatographic column (filled with activated carbon, 6–8 mesh, granular), and glucose residue was identified in the sophorose. Further research on the biological activity of isosteviol was conducted. In addition, a mixture of glucose and sophorose (MGS) was used as the sole carbon source and inducer to increase cellulase production. As a byproduct of the simple stevioside hydrolysis reaction, MGS has a significantly reduced production cost, which is conducive to the industrial production of cellulase, and presents good application prospects. Therefore, we developed an alternative strategy for preparing cellulase inducers *via* a chemical reaction with stevioside as a raw material. It is expected that the results of this study will be of great significance for the biotechnological utilization of lignocellulose.

## Materials and methods

### Microorganisms and medium

The *Trichoderma reesei* Rut C30 was selected for cellulase production in this study, which was kindly donated by the USDA ARS Culture Collection. *Aspergillus niger* ATCC16404, *Saccharomyces cerevisiae* BY4741 and *Escherichia coli* DH5α were used for antibacterial assay. Spores of *T. reesei* Rut C30 and *A. niger* were conserved in cryotubes at −80 °C with 50% glycerol. *S. cerevisiae* BY4741 and *E. coli* DH5α were stored in 30% and 15% glycerol, respectively, at −80 °C for further use.

The medium contained malt extract agar (3 g L^−1^ malt extract and 15 g L^−1^ agar), seed culture medium for enzyme production (5 g L^−1^ glucose and 10 g L^−1^ corn steep liquor) and cellulase fermentation medium (10 g L^−1^ carbon, 1 g L^−1^ peptone, 0.3 g L^−1^ urea, 1.4 g L^−1^ (NH_4_)_2_SO_4_, 2 g L^−1^ KH_2_PO_4_, 0.3 g L^−1^ MgSO_4_·7H_2_O, 0.3 g L^−1^ CaCl_2_, 500 mg L^−1^ FeSO_4_·7H_2_O, 156 mg L^−1^ MnSO_4_·H_2_O, 167 mg L^−1^ ZnC1_2_, 200 mg L^−1^ CoCl_2_, 0.2 mL L^−1^ Tween-80, 500 mL 0.2 mol L^−1^ pH 5.0 Na_2_HPO_4_–citric acid).^31^

### Synthesis of isosteviol and sophorose

Sophorose and isosteviol were prepared according to the method described by Isao Kusakabe *et al.* with slight modifications of the substrate concentration, reaction temperature and reaction time.^32^ The HCl with a concentration gradient (0, 0.04, 0.05, 0.06, 0.07 and 0.08 mol L^−1^) was added into 100 g L^−1^ commercial stevioside (purity ≥ 50%), and the reaction mixture was incubated at 105 °C for 120 min. After the reaction mixture was cooled, the pH of the hydrolysate was adjusted to 5.0 using NaOH, and the mixture was placed at 4 °C for separation and purification. The concentrations of sophorose and glucose were analysed by ion chromatography and biological sensors, respectively, and the concentration of isosteviol was analysed by HPLC.

### Separation and purification

The hydrolysates were separated in a chromatographic column (filled with activated carbon, 6–8 mesh, granular) with methanol as the eluent.^33^ Furthermore, the eluate was examined by high-performance liquid chromatography (HPLC) according to the method described by Uria Bartholomees *et al.* with slight modifications of the elution gradient and excitation wavelength.^34^ The first fraction contained isosteviol, and glucose residue was found in the sophorose in the second fraction. The products of separation were purified in a rotary evaporator (RE-2000A, Yarong, Shanghai, China) at 40 °C and 80 rpm. The separated isosteviol and a mixture of glucose and sophorose (MGS) were used for subsequent microbiostasis experiments and cellulase production after sterilization, respectively.

### Microbiostasis experiment

The bacteriostatic activity of the isolated and purified isosteviol was evaluated using *A. niger*, *S. cerevisiae* and *E. coli*.^35^ Potato dextrose agar (PDA) medium (20 g L^−1^ glucose and 200 g L^−1^ boiled potato), yeast extract–peptone–dextrose (YPD) medium (10 g L^−1^ yeast extract, 20 g L^−1^ peptone and 20 g L^−1^ glucose) and Luria–Bertani (LB) medium (5 g L^−1^ yeast extract, 10 g L^−1^ NaCl and 10 g L^−1^ tryptone) were prepared in 1 mL aliquots, and 2 × 10^4^ spores of *A. niger*, 2 μL of *S. cerevisiae* and *E. coli* at an OD_600_ of 0.5–0.6 were added to the PDA, YPD, and LB media, respectively. During the microbiostasis experiment, three groups, including a negative control group, positive control group and experimental group, were established separately. In the positive control group, 4 μL of hygromycin or kanamycin (50 mg mL^−1^) and 16 μL of distilled water were added to the corresponding medium, and in the negative control group, 20 μL of distilled water was added. In the experimental group, 20 μL of separated and purified isosteviol solution (255.7524701 mg mL^−1^) was added. *A. niger* and *S. cerevisiae* were cultivated at 28 °C and 150 rpm for 20 h, and *E. coli* was cultivated at 37 °C and 180 rpm for 9 h. The bacteriostatic effect of isosteviol was directly observed.

### Production of cellulase

Spores of the fungal strain *T. reesei* Rut C30 were cultured on malt extract agar for 7 d, and the spores were collected using sterile water. For inoculum preparation, one mL of a spore suspension (10^7^ spores per mL) was inoculated into a 250 mL flask containing 50 mL of seed culture medium for cellulase production.^10^ After 24 h of cultivation at 28 °C and 150 rpm, a 4% (v/v) inoculum was added into a 250 mL flask containing 50 mL of fermentation medium, which was modified as described by Mandels *et al.*^14^ After being autoclaved at 121 °C for 25 min, the medium was inoculated with fungal cells, which were then subcultured in an orbital shaker (150 rpm) at 28 °C.^36^ Then, the cells were used to determine filter paper cellulase activity (FPA), biomass, protein concentration, β-glucosidase activity and xylanase activity.

### Enzymatic saccharification in shake flasks

Alkali-pretreatment corn stover (APCS) were prepared as described in our previous study, and the chemical compositions were determined to be 62.6% cellulose, 21.4% hemicellulose, and 8.2% lignin of the dry mass (DM). The crude cellulases induced by MGS as an inducer from *T. reesei* Rut C30 were collected by centrifugation (6000 rpm, 5 min). The enzymes were mixed with APCS and then re-suspended in HAc–NaAc buffer (0.2 M and pH 4.8) with an enzyme loading of 20 FPU g^−1^ APCS and a solid loading of 5% (w/v). Reaction solutions were incubated in a shaking bath at 100 rpm, 50 °C. The samples were collected at different time points, followed by centrifugation at 8000 rpm for 2 min, and the supernatants were subjected to analysis of glucose concentration. While commercial cellulase of Cellic® CTec2 was purchased by Novozymes North America Inc. (Franklinton, NC, USA), β-glucosidase of SUNSON® was purchased from Sunson Industry Group Co., Ltd. (Ningxia, China).

### Analytical methods

Total reducing sugars were analysed *via* the dinitrosalicylic acid (DNS) method recommended by NREL.^37^ The concentration of glucose was determined using a biological sensor, SBA-40 (Institute of Biological, Shandong Academy of Sciences, Jinan, China). The determinations were carried out according to the manufacturers instructions, and each biological replicate was repeated 5 times. Protein concentrations were determined with a Bradford kit (Sangon Biotech, China) using bovine serum albumin (BSA) as a standard. FPA and β-glucosidase activities were determined using the standard protocols reported previously.^38,39^

FPA was determined using filter paper (No. 1, Whatman) as recommended by National NREL Laboratory Procedure LAP006. β-Glucosidase activities were individually determined in 1.0 mL reaction mixtures containing 15 mmol L^−1^ cellobiose dissolved in 0.2 M acetic buffer (pH 4.8). Appropriately diluted enzyme solutions were added after 10 min of incubation at 50 °C. The reducing sugar liberated was measured *via* the dinitrosalicylic acid (DNS) method.^39^ One IU of FPA was defined as the amount of enzyme that released 1 μmol of reducing sugar in 1 min using 50 mg Whatman No. 1 filter paper as the substrate during hydrolysis, whereas one IU of CBA was defined as the amount of enzyme that released 2 μmol of glucose in 1 min using 25 mmol L^−1^ cellobiose as a substrate.

For xylanase activity determination, 180 μL of 1% oat spelt xylan (TCL, Japan) in 50 mM sodium citrate buffer at pH 4.8 was mixed with 20 μL of the diluted enzyme, and the mixture was incubated for 5 min.^38^ The following steps were similar to the cellulase activity analysis. One unit of xylanase activity was defined as the amount of the enzyme needed to release 1 μmol of reducing sugar per minute.

For endoglucanase activity determination, 1 mL of 2% CMC (sodium carboxymethyl cellulose) and 0.5 mL diluted enzyme was added in the test tube and incubated at 50 °C for 30 min. The following steps were similar to the cellulase activity analysis.^39^ One unit of endoglucanase activity was defined as the amount of the enzyme needed to release 1 μmol of berated hydrolysis product per minute.

The concentration of sophorose was determined by the ion chromatography (ICS-5000; Dionex) using the CarboPac PA20 column (Thermo, USA) at the oven temperature of 30 °C equipped with an electrochemical detector (ED40; Dionex). Samples were eluted with acetonitrile and ultrapure water at a flow rate of 1.0 mL min^−1^. The injection volume was 25 μL. Samples were eluted with 0.02 M NaOH at the flow rate of 0.45 mL min^−1^ for 85 min.^10^

The concentration of isolated and purified isosteviol was determined using an HPLC system with a Vertex Kromstar C18 column (250 × 4.6 mm ID, 5 μm particle size). The HPLC system consisted of a Scientific Systems Inc. (SSI) pump and an AT-550 chromatographic column thermostat (Autoscience, Tianjin, China). Samples were transferred to HPLC vials for HPLC analysis using a UV detector (1500; DAD) as described below. The gradient program with acetonitrile as mobile phase A and 20% acetonitrile in water as mobile phase B was as follows: 0–20 min, from 30 to 65% A; 20–20.1 min, from 65 to 30% A; 20.1–30 min, hold 30% A. The separation time was 20.1 min, and the total run time was 30 min. The other conditions included a column temperature of 30 °C, a flow rate of 1.0 mL min^−1^, and a detection wavelength of 210 nm.^34^

The hydrolytic yield of sophorose was calculated as follows:
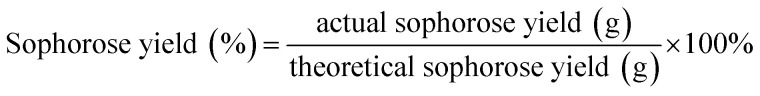


All of the test results were presented as the average values of three parallel tests. The estimated experimental error was used to calculate the “least significant difference” (*p* < 0.05). All data were analysed using GraphPad Prism 8.

## Results and discussion

### Preparation and analysis of sophorose and isosteviol

Stevioside is a good lead compound in the field of medicinal chemistry for diterpenoid drug discovery: it can be hydrolysed to generate the *ent*-kaurane diterpenoid steviol and the *ent*-beyerane diterpenoid isosteviol.^40^ However, there is another hydrolysis product of stevioside that is not used, and if it were to be used as a byproduct, the cost would be low. Therefore, we tried to use this byproduct (a mixture of glucose and sophorose, MGS) to induce cellulase production by *T. reesei* and employed the other hydrolysate, isosteviol, in antibacterial experiments to verify whether it showed biological activity. The optimal acid concentration for stevioside hydrolysis was determined by calculating the efficiency of conversion to sophorose, which is illustrated in Table 1.

**Table 1:** Yields and transformation rates of glucose and sophorose in the presence of different concentrations of HCl

HCl (mol L^−1^)	0	0.04	0.05	0.06	0.07	0.08
Sophorose (g L^−1^)	0	4.36	4.52	4.67	4.5	4.14
Glucose (g L^−1^)	0	28.26	32.84	38.4	40.71	42.02
Sophorose yield (%)	0	15.89	16.76	17.02	16.40	15.09

The highest concentrations of sophorose and isosteviol were observed when stevioside was hydrolysed with 0.06 mol L^−1^ HCl. Under 0.06 mol L^−1^ HCl hydrolysis, the further analysis of MGS by ion chromatography showed that the concentrations of sophorose and glucose were 4.67 g L^−1^ and 38.4 g L^−1^, respectively, while HPLC analysis showed that 6.4 g L^−1^ isosteviol could be obtained. The analysis showed that the concentration of stevioside was 10% under hydrolysis at 105 °C for 120 min with a hydrochloric acid concentration of 0.06 mol L^−1^, and the calculated hydrolytic yield of sophorose was 18.24% (Fig. S1†). In fact, 85.76% of sophorose has been converted (the 14.24% of sophorose was remain in stevioside), but 63.80% of sophorose will continue to be degraded to glucose.

In contrast to MGD, a low glucose concentration is the key to the production of high cellulase levels,^16^ and sophorose can be mixed with a variety of disaccharides to function as an efficient inducer. As a byproduct of stevioside hydrolysis, MGS contains only glucose and sophorose, and the concentration of sophorose is higher than that of MGD. Sophorose is one of the most effective inducers of cellulase production in *T. reesei*.^14^ Therefore, we predicted that MGS would exert an efficient induction effect on cellulase production in *T. reesei*.

A large number of studies have shown that stevioside can be hydrolysed to isosteviol by HCl,^20^ but the effect of the HCl concentration on sophorose conversion has not been studied in depth. In this study, we controlled the concentration of HCl, the only variable, and the data showed that in the presence of 0.06 mol L^−1^ HCl, the conversion rates of sophorose and isosteviol peaked at the same time, which provides a scientific basis for further improving the conversion rate of sophorose, a high value-added product generated in the process of stevioside hydrolysis. Because sophorose can be hydrolysed to glucose by acid, MGS can be obtained, and glucose can inhibit cellulase production by *T. reesei*; therefore, the Rut C30 strain with a *cre1* gene mutation was selected as the enzyme-producing strain in this study. Our previous study also showed that the strain could produce cellulase in the presence of glucose.^41^

### Determination of the bioactivity of isosteviol

**Fig. 1:**
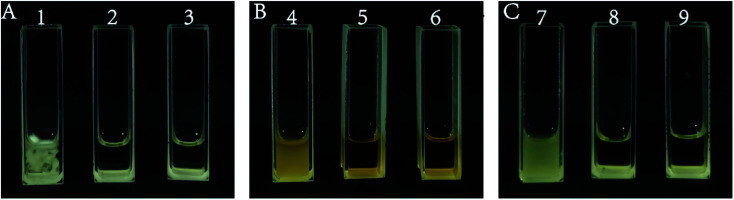
Antibacterial effect on (A) *Aspergillus niger* (ATCC16404) in the negative control group (distilled water), experimental group (isosteviol after separation and purification) and positive control group (hygromycin) (lane no. 1–3); (B) *Saccharomyces cerevisiae* (BY4741) in the negative control group (distilled water), experimental group (isosteviol after separation and purification) and positive control group (hygromycin) (lane no. 4–6); and (C) *Escherichia coli* (DH5α) in the negative control group (distilled water), experimental group (isosteviol after separation and purification) and positive control group (kanamycin) (lane no. 7–9).

Fig. 1 shows the inhibitory effects of isolated and purified isosteviol on *A. niger*, *S. cerevisiae* and *E. coli*. During the microbiostasis experiment, three groups, including a negative control group, experimental group and positive control group, were established. After cultivation in an incubator for the appropriate amount of time, the media of the experimental group (treated with isosteviol) and the positive control group (treated with the corresponding effective antibiotic: hygromycin or kanamycin) had a clear appearance, while the medium of the negative control group (treated with 20 μL of distilled water) was turbid, as shown in Fig. 1. It was concluded from the experimental results that isosteviol had a favourable inhibitory effect on *A. niger*, *S. cerevisiae* and *E. coli*, and the high biological activity of isosteviol was thus verified.

The results showed that the high-value product isosteviol could be successfully obtained by acid treatment with stevioside. The stevioside hydrolysate mixture contained a large amount of isosteviol, and a large number of studies have shown that this compound has excellent biological activity.^25^ Some studies have used *in situ* washing to separate the stevioside hydrolysate to obtain high-purity isosteviol.^42^ In this paper, the chromatographic column separation method was used to separate isosteviol more quickly and easily.

Compared with the biological activity of the isosteviol derivative shown in other studies, the antibacterial effect of purified isosteviol was relatively weak in this study. However, consistent with the present work, some other studies have found that the antibacterial effect of this compound is limited;^28^ therefore, many researchers have tried to modify and transform its structure to enhance its biological activity.^43,44^ Two series of acylthiosemicarbazide and oxadiazole fused-isosteviol derivatives have been synthesized based on the 19-carboxyl modification and demonstrated to show extremely high activity against three cancer cell lines (HCT-116, HGC-27 and JEKO-1).^45^ The structural modification of isosteviol is expected to substantially increase its antibacterial activity, indicating its very strong application prospects. The main purpose of this paper was to investigate whether the byproduct mixture of glucose and sophorose (MGS) could be used as a *T. reesei* inducer, so no further study of the biological activity of isosteviol was conducted.

### Cellulase production by *T. reesei* Rut C30 using different inducers

**Fig. 2:**
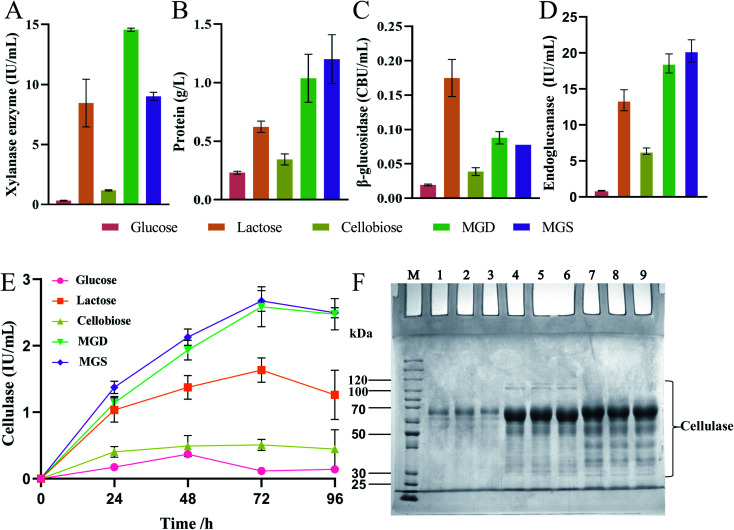
Filter paper cellulase activity, biomass, protein concentration, β-glucosidase activity and xylanase enzyme activity measured in the fermentation broth as a function of time in *Trichoderma reesei* RUT C30 using different inducers (10 g L^−1^ glucose, 10 g L^−1^ lactose, 10 g L^−1^ cellobiose, 10 g L^−1^ MGD or 10 g L^−1^ MGS). The data are presented as the mean and standard deviation of three parallel experiments ((A) xylanase enzyme, (B) protein, (C) β-glucosidase, (D) endoglucanase and (E) cellulase) and SDS–PAGE analysis of cellulase in the fermentation broth of *T. reesei* RUT C30, which was fermented for 60 h with glucose (lane no. 1–3), MGS (lane no. 4–6) or MGD (lane no. 7–9) (F).

To evaluate the cellulase production ability when a mixture of glucose and sophorose (MGS) was used as an inducer, the enzyme activity levels of *T. reesei* Rut C30 obtained using different inducers at the same concentration were compared, as shown in Fig. 2. Even when glucose is present at very low concentrations in the culture medium, it inhibits cellulase production in *Trichoderma* species, except in *T. reesei* Rut C30.^16^ Therefore, based on the above research background, we compared glucose, lactose, cellobiose and MGD as inducers of enzyme production.

Fig. 2A–E shows the enzyme activity of *T. reesei* Rut C30 obtained using different inducers at the same concentration. In the xylanase activity assay, the level of MGS was 38.09% (*p* < 0.01) lower than that of MGD (Fig. 2A). This was due to the greater variety of sugars in MGD than in MGS, which is composed of sophorose and glucose, and the presence of oligosaccharides in MGD, which is conducive to the production of xylanase. At the same time, there was no significant difference in xylanase activity between MGS and lactose. At present, lactose is one of the most widely used soluble cellulase inducers.^12,13^ However, in China, due to the higher cost of lactose than other soluble inducers, lactose has not been applied in the industrial production of cellulase. The xylanase activity of MGS was 27.68- and 7.66-fold higher than those of glucose and cellobiose, respectively (*p* < 0.01). The fermentation broth containing glucose as an inducer showed low enzyme activities, which indicated that although the inhibitory effect of glucose on cellulase production in *T. reesei* Rut C30 was not stronger than that observed in *T. reesei*, its induction efficiency was very low, and it was not suitable as a *T. reesei* Rut C30 inducer.^16^ Cellobiose was associated with low xylanase activity, which indicated that cellobiose presented a low *T. reesei* Rut C30 xylanase induction rate and was not suitable as a cellulase inducer.

Fig. 2B shows that extracellular protein secretion was 5.20-, 1.93-, and 3.49-fold (*p* < 0.01) higher in the *T. reesei* Rut C30 fermentation broth containing MGS than in those containing glucose, lactose and cellobiose, respectively. At this time point, there was no significant difference in the extracellular protein concentration between the MGS and MGD treatments, which indicated that *T. reesei* Rut C30 presented a higher protein production ability under the induction effects of MGS and MGD, and the induction efficiency of MGS and MGD was stronger.

As shown in Fig. 2C, the β-glucosidase activity induced by MGS was 2-fold (*p* < 0.01) higher than that induced by cellobiose, while that induced by lactose was 2.25-fold (*p* < 0.01) higher than that induced by MGS. There was no significant difference in β-glucosidase activity induction between the MGD and MGS treatments. When lactose was used as carbon source, higher β-glucosidase production titer was obtained compared to that with MGS, because β-glucosidase was required to degrade lactose into glucose and galactose to act as substrate for mycelial growth.^46,47^ Besides, β-glucosidase has the ability to catalyze lactose into sophorose by a transglycosidation reaction to efficiently induce cellulase production using lactose as the inducer.^48^ However, hydrolysis and transglycosidation activities of β-glucosidase are not required owing to abundant glucose and sophorose exist in MGS.

Fig. 2D shows the results of endoglucanase production of *T. reesei* Rut C30 using glucose, cellobiose, lactose, MGS or MGD both as substrate for mycelial growth and inducer for endoglucanase production. No significant difference was observed between MGS or MGD, and the endoglucanase activity of MGS was 1.51- and 3.19-fold higher than those of lactose and cellobiose, respectively (*p* < 0.01). Therefore, MGS seems more suitable for endoglucanase production to improve cellulase productivity.

Fig. 2E shows the FPA results of the produced enzymes, which indicated that MGS was the best inducer and a much more efficient carbon source for *T. reesei* than lactose or cellobiose. The levels of FPA were 1.64- and 5.26-fold (*p* < 0.01) higher with MGS than with lactose or cellobiose, respectively. Cellobiose showed no advantage as an inducer of cellulase production, and glucose also showed a marginal induction ability, which was consistent with previous research.^16^ Moreover, there was no significant difference between MGS and MGD, a known efficient inducer of cellulase production by *T. reesei*. This indicated that sophorose in MGS also promoted the transcription of the cellulase gene at a trace concentration, which was equivalent to the β-disaccharide induction level achieved with MGD.^41^ The results showed that MGS, a novel inducer consisting of a sugar mixture, can be used successfully as an inducer in cellulase production; in large-scale processing, MGS would be a better inducer than MGD, and the preparation process would be simpler and more economical.^10,41^

Since there were large amounts of glucose in MGS, glucose was used as a negative control, and MGD was used as a positive control to identify the ability to induce cellulase production. Fig. 2F shows the results of protein secretion by *T. reesei* Rut C30 induced by glucose, MGS and MGD. No significant differences in protein secretion were observed between MGS and MGD, as determined by SDS–PAGE. Based on the small amount of sophorose in MGS, combined with the results of SDS–PAGE analysis, we speculated that the mechanisms by which MGS and MGD induce cellulase production were highly similar.

MGD has been developed for the efficient induction of cellulase production; however, a long high-temperature catalytic reaction is required to obtain MGD, so energy consumption is high, resulting in a high cost of preparation. In contrast, the raw material for MGS, stevioside, is easy to obtain and inexpensive, and MGS can be prepared *via* a simple, rapid process, which undoubtedly makes MGS a more economical cellulase inducer; if the development value of isostevioside increases, the cost of MGS as a byproduct will decrease further. We believe that MGS presents good prospects for use as a low-cost soluble inducer in the industrial production of *T. reesei* cellulase.

### Hydrolysis efficiency of cellulases in *T. reesei*

*T. reesei*, as an industrial fermentation strain of cellulase, has a low level β-glucosidase activity,^49,50^ so the *T. reesei*-derived cellulase (Cel-MGS) by MGS as an inducer was then evaluated for their β-glucosidase cocktail-boosting effect during the hydrolysis of alkali-pretreated corn stover (APCS). SUNSON® was chosen as the β-glucosidase cocktail, as it is known as a highly efficient β-glucosidase activities and lower filter paper and xylanase activity. In addition, the commercially available Cellic® CTec2 enzyme cocktail was included as a reference. The two applied crude enzymes, Cel-MGS and Ctec2, showed large differences in their β-glucosidase, endoglucanase and xylanase activities (Table 2). The specific activities of xylanase and β-glucosidase of Ctec2 were 6.08 and 298.66 times higher than that of Cel-MGS, respectively, indicating robust hemicellulose degradation. However, higher specific activities of cellulase and endoglucanase were obtained when MGS was used as an inducer. This suggests that MGS can provoke high levels of major cellulases production, but low secretion of xylanase and auxiliary activity were achieved by *T. reesei* using MGD as the inducer.

**Table 2:** Specific activities (IU mg^−1^ protein) of the commercial enzymes and in-house generated crude enzymes^a^

Enzyme	Specific activity (IU mg^−1^ protein)			
	Cellulase	β-Glucosidase	Xylanase	Endoglucanase
Cellic® CTec2	0.82	17.92	45.66	12.52
Cel-MGS	2.23	0.06	7.50	16.88
SUNSON®	0.057	1076.37	0.36	0.22

a Cellulase: filter paper unit; β-glucosidase: cellobiase unit.

**Fig. 3:**
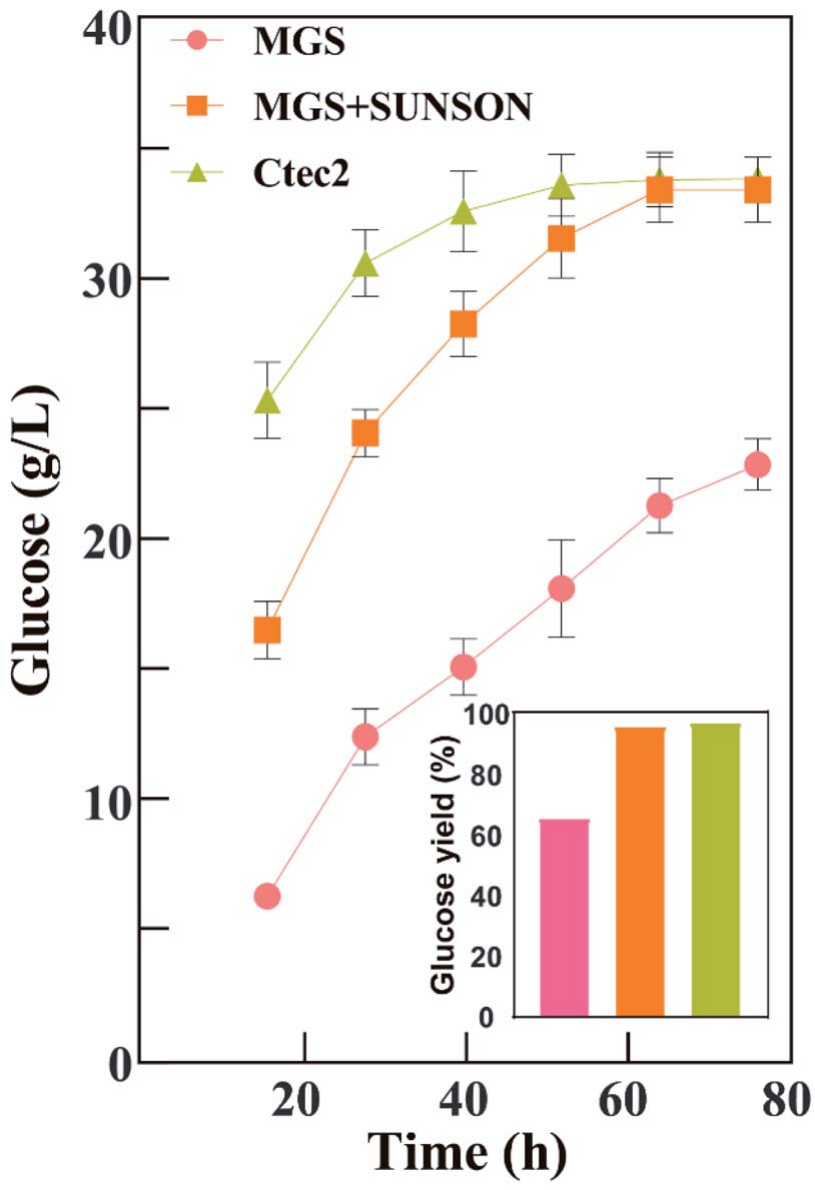
Saccharification of alkali-pretreated corn stover by cellulase from *T. reesei* C30 with MGS, MGS + SUNSON®, and Cellic Ctec2. Hydrolysis was performed at a biomass loading of 5% (w/v) with the same FPase (20 IU g^−1^) at 50 °C and pH 4.8 in a water bath with shaking at 100 rpm.

Fig. 3 shows that glucose concentration was achieved only 22.88 g L^−1^ using Cel-MGS at 72 h with a glucose yield of 65.79%, and the released glucose was improved 30.25% (96.04% yield) using Cel-MGS + SUNSON® compared with the crude enzyme, indicating that improving the β-glucosidase level can relieve the inhibitory effects of cellobiose on exocellulase and endocellulase, thereby significantly improving cellulase activity. Therefore, the cellulase obtained with MGS as an inducer presented great potential for the biotransformation of biomass.

In addition, there was no significant difference in the hydrolysis ability of the cellulase produced under the combined action of Cel-MGS + SUNSON® relative to Cellic® Ctec2, however, the glucose yield of Cellic® Ctec2 was higher than that of Cel-MGS + SUNSON® during the first 24 h. Notably, the β-glucosidase and endoglucanase of MGS + SUNSON® were higher than that of Cellic® Ctec2 (Table 2), indicating that the overall performance in the enzymatic hydrolysis may be attributed to factors other than three major cellulase. Cellulase comprises three major enzyme components: cellobiohydrolases, endoglucanases, and β-glucosidases, along with some other elements involved in cellulose degradation, for instance, the expansin-like protein swollenin (SWO1), GH61 polysaccharide monooxygenases (PMOs) and xylanase, which have been shown to enhance lignocellulose degradation. We believe that the reason for the high hydrolysis efficiency of commercial cellulases is that *T. reesei* cellulase usually includes a variety of these enzymes.^51,52^

Based on the above experimental results, the cellulase obtained by using MGS as an inducer showed a good APCS hydrolysis efficiency. The combined use of MGS and a commercial enzyme could greatly improve the hydrolysis efficiency, and there was no significant difference relative to Cellic Ctec2. The low cost and high efficiency of MGS, a byproduct in the production of isosteviol, will be advantages of its use as an inducer.

## Conclusions

The findings of this study seem to indicate that the mixture of glucose and sophorose (MGS) is a better candidate inducer of *T. reesei* than lactose, cellobiose and a mixture of glucose and β-disaccharide (MGD). MGS, as a byproduct, is a low-cost inducer of cellulase production in *T. reesei*. MGS, acting as a soluble inducer, induced cellulase production in *T. reesei* more strongly than cellobiose or lactose. Although there was no significant difference in induction efficiency between MGS and MGD, MGS showed a lower production cost and simpler preparation process than MGD, because it was a byproduct of stevioside hydrolysis. This study not only provides a novel method for preparing inducers with low cost but will also benefit research on improving cellulase production using soluble inducers for easier operation in industrial-scale cellulase production. Isosteviol, which is one of the hydrolytic products of stevioside, shows extensive bioactivity according to microbiostasis experiments and is a high-value-added product. The synthesis and biological activity of isosteviol are described in this paper for scholars engaged in related research on isosteviol.

## Author contributions

Peng Zhang: writing-original draft, methodology, data curation and visualization. Qian Li: visualization and editing. Yudian Chen: data curation and editing. Nian Peng: validation and methodology. Wenshu Liu: formal analysis and validation. Xuemei Wang: data curation and methodology. Yonghao Li: conceptualization, supervision, writing-review and editing.

## Conflicts of interest

The authors declare that they have no known competing financial interests or personal relationships that could have appeared to influence the work reported in this paper.

## Supplementary Material

RA-012-D2RA01192A-s001
