# Supplementary figures and images for: Induction of cellulase production in Trichoderma reesei by a glucose–sophorose mixture as an inducer prepared using stevioside
Source: RSC Adv. 2022 Jun 13;12(27):17392–400. doi: 10.1039/d2ra01192a (PMC9190947; doi:10.1039/d2ra01192a)

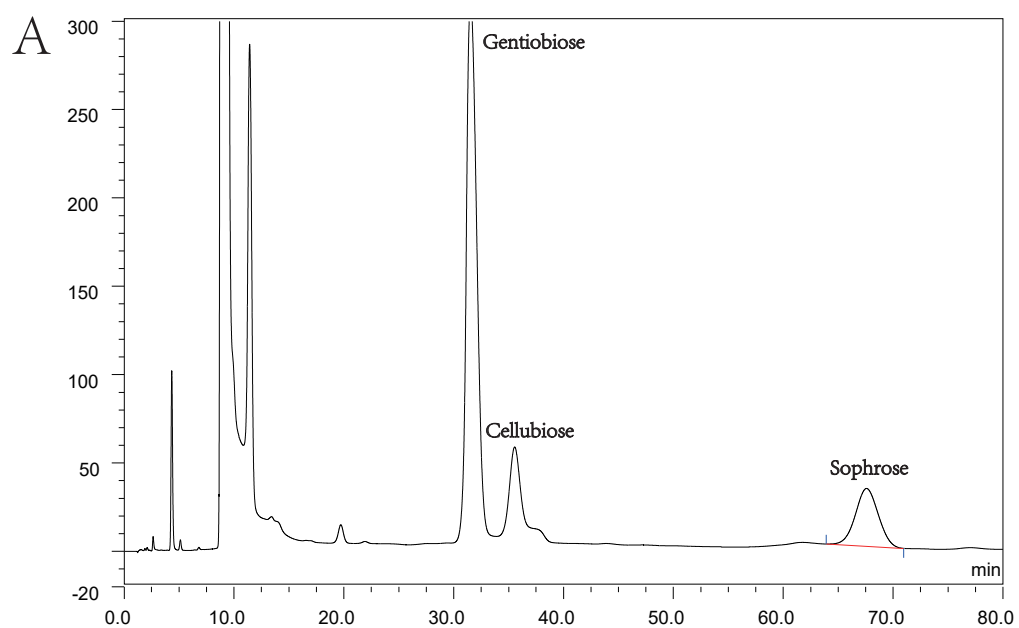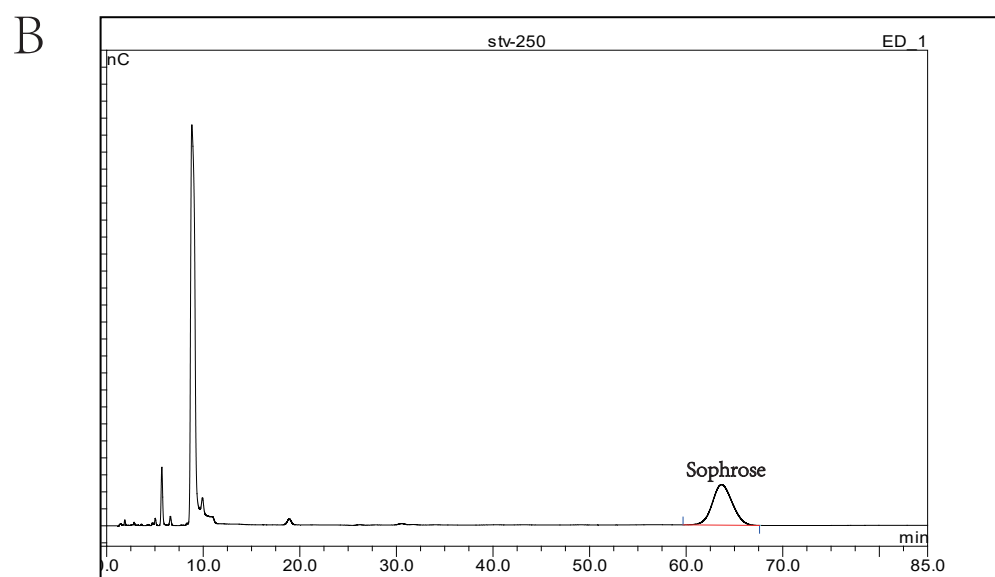

Supplement: RA-012-D2RA01192A-s001 [file RA-012-D2RA01192A-s001.pdf]
